# Primary huge gastric leiomyosarcoma with multiple metastases in a 60-year-old female: a case report

**DOI:** 10.11604/pamj.2022.42.223.35513

**Published:** 2022-07-21

**Authors:** Fayed Al-Yousofy, Gamal Alshargabi, Faisal Ahmed, Abdullatif Almohtadi, Muneer Fazea, Abdulfattah Altam

**Affiliations:** 1 Department of Pathology, Faculty of Medicine, Taiz University of Medical Sciences, Taiz, Yemen,; 2 Department of General Surgery, School of Medicine, Taiz University of Medical Sciences, Taiz, Yemen,; 3 Urology Research Center, Al-Thora General Hospital, Department of Urology, School of Medicine, Ibb University of Medical Sciences, Ibb, Yemen,; 4 Department of Radiology, Ibb Scan Center, Ibb, Yemen,; 5 Department of Radiology, School of Medicine, Ibb University of Medical Sciences, Ibb, Yemen,; 6 Department of General Surgery, School of Medicine, 21 September University, Sana'a, Yemen

**Keywords:** Primary leiomyosarcoma, stomach, pathology, case report

## Abstract

The incidence of leiomyosarcomas (LMS) has declined drastically. In fact, the introduction of immunohistochemistry (IHC) helped to differentiate LMS from other gastrointestinal stromal tumors (GIST) by receptor tyrosine kinase (KIT)-mutation detection making gastric LMS a sporadic tumor recently. We report a 60-year-old female who presented with a three-week history of abdominal pain. An abdominal computed tomography scan showed a large exophytic mass (22 ×19 ×15 cm) arising from the greater curvature of the stomach with multiple metastases. A biopsy was taken, and the initial histopathological examination was suggestive of GIST. However, further histopathological examination confirmed a high-grade gastric LMS. The patient refused any surgical intervention. Therefore, the patient had only received chemotherapy. On 9-month follow-up, the patient is still alive without disease progression. In conclusion, gastric LMS is a rare tumor. Due to the possibility of being misdiagnosed with other GIST, extensive pathological evaluation through specialized experts and IHC analysis is recommended.

## Introduction

Sarcomas are cancerous mesenchymal malignancies that make up less than 1% of all adult solid hematological malignancies [1]. The histopathology of the leiomyosarcoma (LMS) is similar to the gastrointestinal stromal tumor (GIST), in fact, many GISTs cases were previously diagnosed as LMS; however, with the implementation of KIT mutation immunostaining, which is the most common genetic changes associated with GIST, they become a less frequent entity with a prevalence of 1% [2,3].

Because this tumor's growth is often insidious, and it is usually discovered at an advanced stage. As a result, knowledge of the biology and scientific behavior of LMS in the stomach is restricted, and the presentation of new cases is vastly necessary [4,5]. We present a 60-year-old female diagnosed with gastric LMS with multiple metastases, focusing on its clinical manifestations, diagnosis, and treatment.

## Patient and observation

**Patient information:** a 60-year-old woman with morbid obesity presented to our outpatient clinic with a chief complaint of mild abdominal pain and left lower extremity edema that started three weeks ago. Her abdominal pain was localized in the epigastric area and radiated to the left lower quadrant, puncturing type, worsened with lying down, and not relived with analgesic therapy. The patient had a history of chronic anemia that started six months ago without response to medication or blood product transfusion. The patient had diabetes mellitus type 2 on oral hypoglycemia drug agents. No history of melena, jaundice, fever, vomiting, nausea, or urinary tract symptoms was mentioned. Her family had no history of malignancy or other chronic medical illness.

**Clinical findings:** in physical examination, the patients' vital signs were blood pressure: 100/70 (mmHg), respiratory rate: 14 (respirations per minute), pales rate: 61 (beats per minute), oral temperature: 37.5°C, and body mass index (BMI): 35 kg/m^2^. She was pale, and an abdominal exam revealed a mildly distended abdomen with palpated abdominal mass in the upper and lower left quadrant with mild tenderness. There was mild pitting edema in both lower extremities.

**Figure 1:**
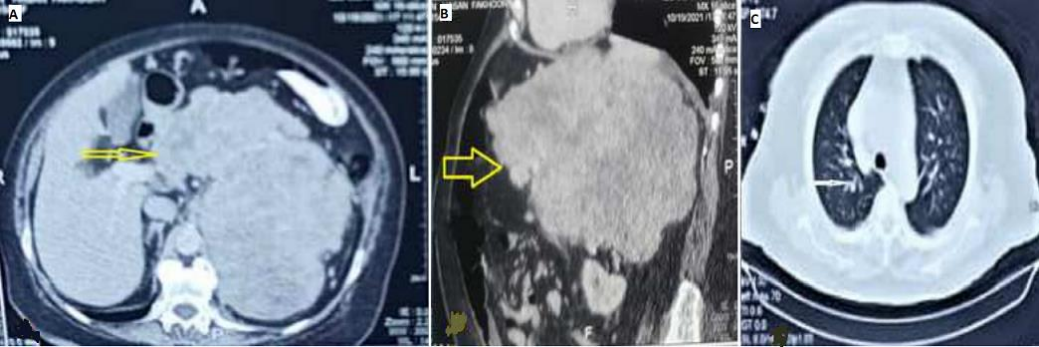
A) axial; B) sagittal views of abdominal CT scan revealing a large mixed echogenic mass arising from the gastric wall involving the surrounding organs (arrow); C) showing lung metastasis (arrow)

**Diagnostic assessment:** the white blood cells: 11 ×10^3^/ml, hemoglobin: 7 g/L, platelets count: 200 × 10^3^/L, reticulocyte's count: 9.5%, erythrocyte sedimentation rate (ESR): 24 mm/hr, and serum ferritin: 10 ng/L. The stool exam was positive for occult blood. The other blood investigation tests, including liver function tests, coagulation tests, and renal function tests, were within normal limits. Abdominal and chest computed tomography (CT) scan showed a 22 ×19 ×15 cm ill-defined, heterogeneous, lobulated mass with main bulk in the left hypochondrium, arising from the gastric wall (greater curvature). The mass cross the midline causing displacement of the aorta and the celiac artery, and had a pressure effect on the pancreas, stomach, spleen, and bowel loops. There were multiple metastases of the lung, liver, and multiple pulmonary and mesenteric lymphadenopathies (Figure 1 A,B).

**Figure 2:**
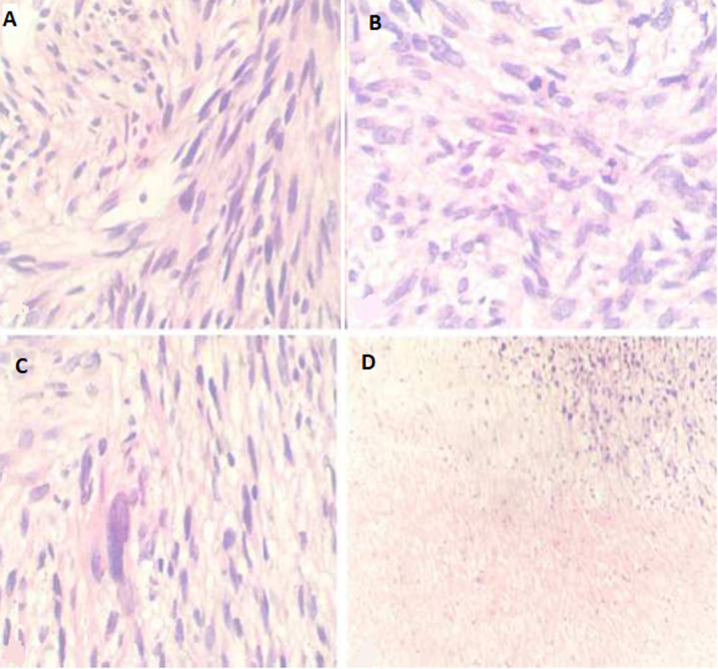
A) the tumor showing a fascicular growth pattern of smooth muscle cells with cigar-shaped nuclei; B) abnormal mitosis; C) pleomorphism; D) and massive coagulative type necrosis

**Figure 3:**
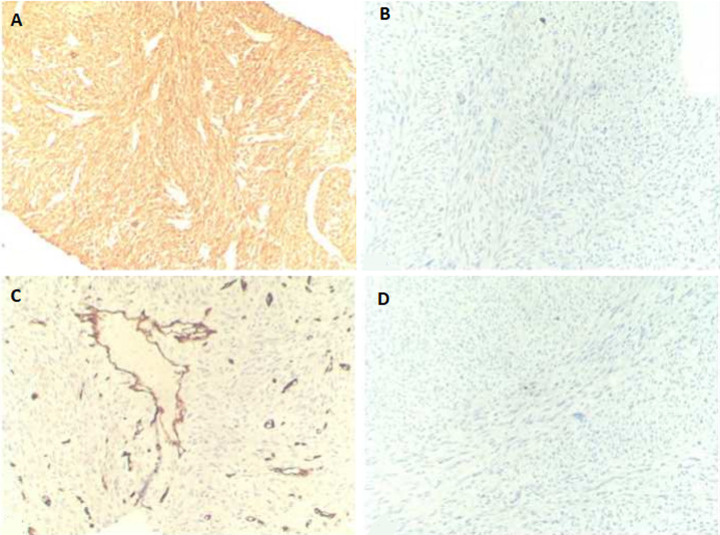
A) immunohistochemistry showing that tumor cells are positive for SMA; B) negative for CD117; C) CD34; D) and S100

The ultrasonography (US) guided true-cut biopsy from the abdominal mass was performed. The initial histopathology result was highly suspected of GIST. The histopathology was reviewed by an expert pathologist, which showed a fascicular growth pattern of smooth muscle tumor and nuclear pleomorphism suggestive of a high-grade gastric LMS (Figure 2 A,B,C,D). Immunohistochemistry (IHC) analyses revealed fascicular spindle cell neoplasm with brightly eosinophilic cytoplasm and cigar-shaped nuclei; this high-grade tumor exhibits significant cytological pleomorphism. Tumor cells expressed were positive for SMA and negative for CD34, CD117, and S100 (Figure 3 A,B,C,D).

**Therapeutic interventions:** the patient has rejected any surgical interventions. For that, the multidisciplinary team recommended the treatment with chemotherapy agents with paliation intent. The patient received five cycles of doxorubicin (90mg/cycle) in monotherapy but showed disease progression after five months of chemotherapy with increasing size of the mass and the number of hepatic lesions. Subsequently, the chemotherapy was changed into six cycles of gemcitabine-docetaxel combination, starting with gemcitabine (1400 mg) on day one, followed by a combination of gemcitabine (1200 mg) with docetaxel (100 mg) on day 8.

**Follow-up and outcome:** within nine months of follow-up, the patient was still alive, had no disease progression, and with an acceptable condition.

**Patient perspective:** during treatment, the patient was satisfied with the level of care provided to her.

**Informed consent:** written informed consent was obtained from the patient for participation in our study.

## Discussion

Leiomyosarcoma (LMS) accounted for nearly 5%-10% of all soft-tissue sarcomas and are most commonly found in the uterus, digestive system, and retroperitoneal region. Since Hirota *et al*. first described GIST with C-kit mutations, gastric LMS has been rarely mentioned and accounts for 1% of all malignant gastric tumors [2,6]. The cause of LMS is unknown, but exposure to radiation, chemical substances, Epstein-Barr virus, and low immunity may be risk factors for this malignancy [7].

Radiologic images such as the ultrasound scan (US), CT scan, and magnetic resonance imaging (MRI) can help in LMS diagnosis. However, they cannot provide a definitive diagnosis. The US has a lower-cost and used to evaluate the tumor size, location, and cystic versus solid lesions. Nevertheless, it is ineffective for follow-up tumor sizes after treatment [8]. The primary imaging technique for assessing and evaluating LMS and the existence of metastatic disease is the CT scan [9]. MRI can provide anatomical identification of the tumor' location, attachment, and adhesion of cancer with the adjacent blood vessels and structures [7]. In our patient, the mass was discovered incidentally, which was enormous in the CT scan, crossed the midline, and causing displacement of the main arterial blood vessels with a pressure effect on the gastrointestinal organs nearby.

Microscopically, LMS should be distinguished from other spindle cell tumors, especially GIST and nerve sheath tumors (schwannomas and malignant peripheral nerve sheath tumors). Distinction from benign LMS is mitosis (>10/50HPF), atypia, and coagulative necrosis, which is found in LMS and absent in benign leiomyoma. The primary differential diagnosis histologically is GIST, which should be notably excluded in all gastric cases. In our case, the initial histopathology result was highly suspected gastrointestinal stromal tumors. The histopathology was reviewed by an expert pathologist, which showed a high-grade gastric LMS. A similar report was mentioned by Garg *et al*. [2]. GIST should be positive for at least two markers of the three CD117, CD34, and Dog-1 [3,10,11]. In our case, the GIST was excluded by negative CD117 and CD34; neurogenic tumors were excluded by S100 negativity.

Gastric LMS is a cruel disease that does not respond to chemotherapy or radiation [12]. Only surgery is curable, and half of the patients will experience local recurrence and distant metastases after tumor resection. Doxorubicin and ifosfamide are first-line treatments for sarcomas, but they are ineffective for gastric LMS, with a response rate of only 18% [13]. Although, gemcitabine and docetaxel are effective adjuvant treatments for uterine LMS; however, their efficacy for gastrointestinal LMS has not been demonstrated [12,14]. A recent study by Kawaguchi *et al*. reported that gemcitabine and doxorubicin could regress the LMS in the patient-derived orthotopic xenograft model [12]. Additionally, Penel *et al*. retrospectively studied 147 adult patients with metastatic LMS. The authors concluded that maintenance of the doxorubicin alone was associated with improved progression-free survival, and combined chemotherapy regimens did not improve the outcome [15].

In literature, the benefits of radiotherapy in addition to surgery are controversially discussed. While Nussbaum *et al*. reported that both neoadjuvant and adjuvant radiotherapy was associated with improved 5-year-survival compared with surgery alone [16]. Hager *et al*. in the multivariate analysis, showed that the overall survival was not affected by receiving radiotherapy [17].

Leiomyosarcoma (LMS) has a propensity for hematogenous spread. Distant metastases are present in approximately 40% of diagnosed patients, and most patients finally develop metastasis [7,18]. Additionally, the presence of metastases at the time of diagnosis, and tumor size of more than 5 cm are associated with poorer-prognosis [2]. The overall 5-year survival rate for patients with gastric sarcomas varies and has been reported to range between 16% and 56%. It is determined by the tumor's grade and the success of complete surgical resection. Recurrences typically occur two years after resection and occur in 36% to 60% of cases [2]. Although the liver and lungs are the most common locations of metastasis, LMS has also spread to the pancreas, small bowel, cardiac chambers, skin, submandibular salivary gland, scalp, skeletal muscles, and subcutaneous tissue [7]. Mehta *et al*. reported a 47-year-old man with a gastric LMS and metastasis to the liver treated with doxorubicin and a docetaxel-gemcitabine combination and stable disease achieved with pazopanib [7]. Our patient was morbidly obese, had large unresectable tumors with lung and liver metastasis, and the patient was refused the surgery. The chemotherapy was started, and the patient was still alive within nine months of follow-up with an acceptable condition.

## Conclusion

Gastric LMS is a rare cancer. Histopathological examination of the specimen with IHC analysis is the optimal choice for making an accurate diagnosis. Due to the possibility of being misdiagnosed with other gastrointestinal stromal tumors, extensive pathological evaluation through specialized experts and IHC analysis is recommended.
